# Supplementary material for: Content-rich biological network constructed by mining PubMed abstracts
Source: BMC Bioinformatics. 2004 Oct 8;5:147. doi: 10.1186/1471-2105-5-147 (PMC528731; doi:10.1186/1471-2105-5-147)
Supplement: Additional File 5 — The original Chilibot query results of the term "long-term potentiation (LTP)" and 22 other terms, limiting the latest references analyzed to the years 1990, 1995, 2000, and 2004. [file 1471-2105-5-147-S5.bz2 › chilibotAdditionalFile5/ltp2000/html/PLC.html]

 


**PLC** (Input: PLC ) 

---


**Google Searches:** Entire Web  | EDU domain only  | PDF files only

.

**External Links:** OMIM |
LocusLink
| Swissprot
| GeneCards

  
**Maps of PLC**

Simple
Complete
graph in
radiant
tree
square
layout.

**New Hypothesis !**

**Synonyms** 

- plc   [PubMed]

**Synopsis**

- These results suggest that two different pathways, the c ras and the **PLC** gamma pathway, are activated by TrkB receptors in primary neurons.  J Neurochem, 1995 
  [23]

- These results, together, suggest that a hyperosmotic NaCl induced signaling pathway that leads to activation of ERK1 2 may sequentially involve **PLC** activation, DAG release, and cPKC and nPKC activation.  Am J Physiol Cell Physiol, 2000 
  [20]

- Since Grb2 binding is NOT affected by this mutation, these results strongly suggest that **PLC** gamma activation regulates Ras activation in these cells.  J Biol Chem, 2000 
  [20]

- Finally, our study suggests that PC **PLC**,   Biochem J, 2000 
  [19]

- Using immunoprecipitation, PI3 kinase and tyrosine phosphorylated **PLC** gamma1 and SHC were shown to be associated with tyrosine phosphorylated TrkB in response to both BDNF and NT 4.  Exp Neurol, 1999 
  [18]

- Finally, inhibition of PI **PLC** results in NGF s ability to stimulate DNA synthesis in the absence and presence of IGF I.  Oncogene, 1999 
  [16]

- These observations suggest that the 1S,3R ACPD induced burst firing is NOT mediated by **PLC** coupled metabotropic glutamate receptors.  Neuropharmacology, 1994 
  [16]

- These changes in calcium dependent PLA 2 and **PLC** beta 1 activities, coupled with reports of FAE induced deficits in protein kinase C activity, indicate that prenatal exposure to moderate quantities of ethanol causes profound and long lasting deficits in the cellular signaling mechanisms associated with activity dependent synaptic plasticity and memory formation.  Alcohol Clin Exp Res, 1997 
  [16]

- Moreover, PD98059 inhibited the expression of cytokines induced by LPS or PC **PLC**, thus suggesting that ERK activity is required for macrophage activation by these two agents.  J Biol Chem, 2000 
  [16]

- Second, synergy of IP accumulation in correlation with synergy of neurotransmitter release elicited by mAChR activation and membrane depolarization, suggests a possible role for phospholipase C **PLC** in the bifurcating control of neurotransmitter release and for the involvement of **PLC** and voltage sensitive channels in mediation of long term potentiation  [LTP]  LTP .  Neurosci Lett, 1990 
  [16]

- After NGF treatment, NGF was bound to TrkA in endocytic organelles, and TrkA was tyrosine phosphorylated and bound to **PLC** gamma1, suggesting that these receptors were competent to initiate signal transduction.  J Neurosci, 1996 
  [15]

- These results indicate that G i o protein, tyrosine kinase, P13K, PKC, and **PLC** calcium,   FEBS Lett, 1999 
  [14]

- The evidence presented is consistent with the hypothesis that **PLC** acts as a coincidence detector in LTP.  Hippocampus, 1998 
  [12]

- Thus, activation of TrkB and **PLC** leads to a TRPC3 dependent cation influx in CNS neurons.  Neuron, 1999 
  [12]

- These results indicate that in developing cortical neurons, activation of AMPA KA receptors by KA may induce expression, followed by activation of TrkA via **PLC** signaling and intracellular calcium elevation and hence increase reception of NGF on KA challenged neurons.  J Neurochem, 2000 
  [11]
